# Diffuse Myocardial Calcifications Caused by Leptospirosis

**DOI:** 10.7759/cureus.45345

**Published:** 2023-09-16

**Authors:** Vesna Lesjak, Maja Pirnat

**Affiliations:** 1 Radiology, University Medical Centre Maribor, Maribor, SVN

**Keywords:** leptospira, diffuse myocardial calcifications, multi-parametric mri, sepsis, leptospirosis

## Abstract

Severe leptospirosis is defined by multiple organ failure. Cardiac involvement is an uncommon complication in patients with leptospirosis, and the pathophysiology of it is not well understood. Diffuse myocardial calcifications connected with sepsis are infrequent, and their effect on heart function is hard to predict. They can lead to conduction disorders and arrhythmias, thereby causing sudden death. Myocardial calcifications are usually revealed incidentally by radiological investigations such as computed tomography (CT) scan in patients with or after sepsis and are commonly unidentified in practice because most cases progress gradually. This case report involves a 51-year-old male who presented to the emergency department with sepsis. The patient was diagnosed with leptospirosis, causing septic cardiomyopathy and diffuse calcifications of the myocardium of the left ventricle. This case highlights the importance of multimodality imaging and a multidisciplinary approach to diagnoses since early recognition and treatment are essential. Follow-up of such patients is necessary to monitor the systolic function of the left ventricle and cardiac arrhythmia.

## Introduction

Leptospirosis is a well-known zoonosis with significant morbidity and mortality. Cardiac involvement is a rare complication, although dysfunction of the heart is a common complication of severe sepsis and shock [1]. Myocardial calcifications are described as dystrophic when they occur in injured myocardium in a patient with normal calcium homeostasis and as metastatic when they occur in intact myocardium in a patient with abnormal calcium homeostasis [2]. We present the case of a middle-aged male who presented with severe leptospirosis with cardiac involvement and developed diffuse myocardial calcifications.

## Case presentation

A 51-year-old previously healthy male was presented to the emergency department with complaints of fever, headache, myalgia, diarrhea, and shortness of breath that had persisted for three days. The patient denied recent travel, sick contacts, or participation in adventure sports. Upon admission, the patient was hypotensive (blood pressure 88/65 mmHg), tachycardic (heart rate 124 beats/min), and subfebrile (temperature 37.5°C); he was in a state of shock. The electrocardiogram showed sinus tachycardia without ST-T abnormalities. He had acute renal failure with elevated serum creatinine (505 µmol/L) and a glomerular filtration rate of 11 mL/min/1.73 m^2^. Laboratory results showed an elevated white blood cell count (21.5×10^9^/L), as well as highly elevated C-reactive protein (474 mg/L) and troponins (high sensitivity troponin I (hsTNI) 9860 ng/L). He had elevated lactate levels (11.9 mmol/L), hemoglobin level was normal. At this time, a preliminary diagnosis of severe sepsis with septic cardiomyopathy was established. After ICU admission, a polymerase chain reaction (PCR) was positive for leptospirosis in serum and urine.

**Figure 1:**
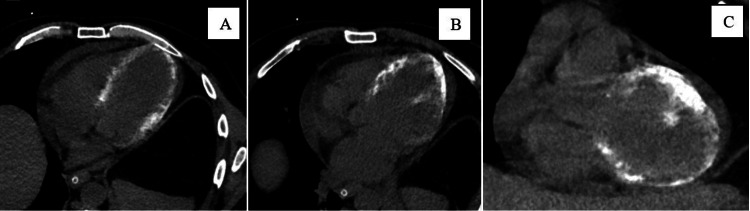
Non-contrast CT scan reveals the extensive myocardial calcifications in the LV wall. (A,B) Soft-tissue window, four-chamber view. (C) Soft-tissue window, short-axis view. CT: computed tomography; LV: left ventricular.

**Figure 2:**
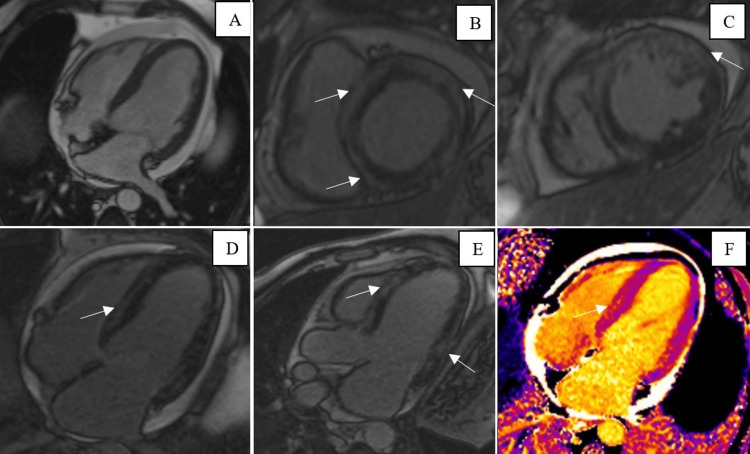
(A) Balanced short-axis steady-state free precession CMR, four-chamber view denotes dilated left ventricle size and pericardial effusion. LGE on short axis (B, C), midventricular four-chamber view (D), and three-chamber view (E) demonstrates diffuse basal to apical predominantly subepicardial enhancement (arrows). (F) Mid-left ventricular long-axis native T1 map demonstrates increased T1 relaxation time in the corresponding regions of late gadolinium enhancement (arrow). Qualitatively, the region of interest is brighter on the color map compared to the surrounding myocardium. CMR: cardiovascular magnetic resonance.

Initial transthoracic echocardiography (TTE) on admission to the ICU showed left ventricular ejection fraction (LVEF) of 25% and no pericardial effusion. A computed tomography (CT) scan performed 20 days after hospital admission revealed an increased density in the myocardium of the left ventricle with myocardial calcifications in the apex, septum, anterolateral and inferior wall, and anterior papillary muscle (Figure 1), as well as some pericardial effusion. Follow-up TTE prior to discharge showed restored systolic function with an LVEF of 55% and hemodynamically insignificant pericardial effusion. The right ventricular function remained normal. Mild aortic and mitral regurgitation were noted at baseline and at follow-up. Cardiovascular magnetic resonance (CMR) imaging was performed on a 1.5 Tesla MRI 10 weeks after admission. On cine CMR, there was mild myocardial thickening corresponding to the areas of calcification observed on the CT. CMR showed a dilated LVEF of 42%; right ventricular function was normal. Pre-contrast mapping showed higher T1 relaxation time (1254 ms) and T2 values (58 ms) on mapping sequences. Late gadolinium enhancement (LGE) imaging showed diffuse delayed enhancement corresponding to the areas of calcification observed on the CT (Figure 2).

A final diagnosis of septic cardiomyopathy caused by leptospirosis was made on the basis of a combination of clinical, laboratory, and imaging findings. This was further affirmed by the rapid appearance of myocardial calcifications and the presence of normal serum calcium levels in the course of hospital admission.

He was discharged after two months with heart failure medications, including beta-blockers and angiotensin-converting enzyme inhibitors, to manage his reduced cardiac function. The patient’s symptoms gradually improved; on follow-up 12 months after discharge, he reported a reduction in fatigue, improved exercise tolerance, and underwent an ultrasound (TTE), which showed normal LV size, restored systolic function of LV, and no pericardial effusion. The right ventricular function was normal. 

## Discussion

Leptospirosis is a zoonotic disease caused by pathogenic spirochetes of the genus *Leptospira*. It is a rare infection, although the most widespread zoonosis, and is reappearing around the globe [1]. It is a disease that has two distinct phases: an early septicemic phase that lasts for a week and a delayed immune phase. In the acute phase, the patients present with chills, fever, muscle and abdominal pain, conjunctival suffusion and photosensitivity, nausea, diarrhea, and vomiting. Most complications develop in the immune phase of the disease, which occurs in less than 10% of patients. The vast majority of infections are subclinical and resolve naturally. Nonetheless, in some cases, the infection progresses to the icteric phase (“Weil’s disease”) and manifests as kidney failure, jaundice, pulmonary hemorrhage, acute respiratory distress syndrome (ARDS), myocarditis, rhabdomyolysis, or septic shock. It can also involve the CNS. The mortality rate ranges from 3% to 5% in severe cases and is predominantly caused by acute kidney or liver failure, myocarditis, pulmonary hemorrhage, and multiorgan failure [1,3]. The pathophysiology of cardiac involvement in leptospirosis is not well understood [4]. Studies suggest that involvement of the heart is frequent and probably underestimated [5]. Autopsy studies report important cardiac involvement in fatal leptospirosis [6]. The presence of myocarditis is reported beyond the first five days to one week of illness, and severe cardiac dysfunction in patients is rare [4].

Leptospirosis can lead to various, usually transient, ECG abnormalities, such as atrial fibrillation, bundle branch conduction blocks, and non-specific ventricular repolarization disturbances. Abnormal serum electrolyte levels (of magnesium, calcium, sodium, and potassium) are likely to contribute to ECG changes [7]. Histopathological changes in the myocardium have been shown in autopsy studies, with myocardial inflammation and vasculitis, but the pathophysiology of cardiac involvement in leptospirosis is inadequately understood [1]. Cardiac involvement, demonstrated clinically or electrocardiographically, predicts poor outcome [1,8].

Our patient developed extensive myocardial calcifications, which were caused by severe leptospirosis. Myocardial calcification can appear for a lot of reasons. Two basic forms are recognized: metastatic and dystrophic. Metastatic calcification occurs as a consequence of systemic process, i.e., hypercalcemia or abnormalities of calcium homeostasis, and can appear in normal or unhealthy tissue. Metastatic calcifications are usually found in patients on hemodialysis (with chronic kidney failure), but they have also appeared in patients with oxaluria, primary, secondary, or tertiary hyperparathyroidism, and a nutritional deficit of calcium and vitamin D [2,9]. Different etiologies of dystrophic myocardial calcification have been described. Most commonly, they occur after previous myocardial infarction; other causes are traumatic (after operation, irradiation), inflammatory (rheumatic heart disease, sarcoidosis, sepsis), infectious (myocarditis, myocardial abscess), neoplastic processes, or after administration of certain drugs (including cyclosporine, steroids, etc.). This type of calcification is the result of local tissue damage and cell necrosis. The etiology of myocardial calcification can also be multifactorial. Also, a number of inflammatory processes can affect both the heart and the kidney, resulting in dystrophic calcification aggravated by abnormal calcium metabolism [2]. Myocardial calcifications can develop days to weeks after sepsis onset [9].

Sepsis-related myocardial injury is a very complex process [10,11]. In state of sepsis, dystrophic calcification may take place as a consequence of myocardial injury caused by several cardiotoxic factors, such as oxidative stress, inflammatory cytokines, microvascular ischemia, and excess catecholamine (external or internal). The incidence in septic patients is unexplored but appears to be extremely uncommon since only a few case reports have been documented [9,12]. Our patient had normal serum calcium levels throughout the hospitalization, despite the sepsis and calcifications developing rapidly.

CT scan is the gold standard for the noninvasive detection of calcifications, and the diagnosis of myocardial calcification is usually unexpected, achieved accidentally by thorax CT [12]. In our patient, it exposed the abundant myocardial calcifications in the myocardium of the left ventricle and the less noticeable calcifications in the anterior papillary muscle. Echocardiography is less sensitive at revealing calcifications within the myocardium but gives additional information about heart function since hypokinesia and diastolic dysfunction can be present. Nevertheless, echocardiography can be normal or almost normal. CMR does not routinely contribute to diagnosis. In the case of myocardial calcification due to sepsis, the calcifications are located in the necrotic myocardium as well as the interstitial space, and this leads to increased extracellular space. Increased extracellular space subsequently leads to hyperenhancement since regional variations in cardiac extracellular volume and various patterns of absorption and washout within the extracellular space are the causes of late gadolinium enhancement. Calcifications make no signal or a weak signal on CMR and appear dark on all sequences, together with LGE. In the setting of acute myocardial calcifications, the calcified myocardium is assembled from necrotic myocardium with calcium deposits in the myocardium and in the interstitial space, leading to gadolinium enhancement that imitates fibrosis [13].

The definition of sepsis-induced cardiomyopathy (SIC) has been summed up as a global and reversible heart dysfunction [14-17]. A retrospective study reported that it developed in 13.8% of patients with sepsis and septic shock [15]. In our patient, the reduced EF at the time of admission was probably due to sepsis-induced cardiomyopathy. One of the characteristics of SIC is recovery in seven to 10 days [14]. The LVEF normalization was also seen on follow-up TTE. At the CMR that was performed 2.5 months after presentation, LVEF was reduced (42%). This could be due to the progression of diffuse myocardial calcifications, and therefore, the follow-up of such patients should be obligatory.

This case emphasizes the importance of using advanced imaging techniques like multi-parametric MRI to aid in diagnosis. Collaboration between infectious disease specialists, cardiologists, and radiologists is vital for comprehensive patient care and appropriate management.

## Conclusions

Myocardial inflammation and involvement in leptospirosis can go unacknowledged due to non-specific clinical findings and co-concomitant multi-organ dysfunction. Sepsis itself causes cardiomyopathy, characterized by ventricular dilatation and a decrease in EF. Diffuse myocardial calcifications related to sepsis caused by leptospirosis are an extremely uncommon phenomenon but can lead to greater morbidity and mortality. These calcifications are usually abundant and involve the LV wall or, less frequently, the right ventricular wall or papillary muscles. Myocardial calcification is usually an incidental finding on a CT in patients with, or after, sepsis and is unrecognized and underdiagnosed in everyday practice since most cases progress quietly; therefore, it is important to use advanced imaging techniques like multi-parametric MRI to aid in diagnosis. Regular follow-up is mandatory for surveillance of the systolic function of the left ventricle and arrhythmia.
